# The utility of fragmented QRS in association with strain echocardiography in predicting significant coronary artery stenosis

**DOI:** 10.34172/jcvtr.33253

**Published:** 2024-12-23

**Authors:** Seyed Abdolhossein Tabatabaei, Elham Omrani, Atoosa Mostafavi, Hakimeh Sadeghian, Ali Abbasi

**Affiliations:** Department of Cardiology, Dr. Shariati Hospital, Tehran University of Medical Science, Tehran, Iran

**Keywords:** Strain, Fragmented QRS, Coronary angiography, Coronary artery disease

## Abstract

**Introduction:**

Fragmented QRS (fQRS) on a 12-lead ECG indicates electrical conduction disruption due to various cardiac issues, including coronary artery disease (CAD). This study investigated whether combining fQRS and reduced myocardial strain could predict significant CAD.

**Methods:**

We conducted a cross-sectional study on patients with fQRS on surface ECG who underwent coronary angiography. The left ventricular strain was assessed using 2D speckle-tracking echocardiography.

**Results:**

We enrolled 55 patients with fQRS and significant CAD (≥70% coronary artery stenosis) and 55 control patients (≤30% stenosis). The strain was significantly reduced in segments with fQRS and significant CAD compared with the control group.

**Conclusion:**

In patients with CAD, the combination of fQRS in any ECG lead and reduced strain can predict the presence and location of a coronary artery with greater than 70% stenosis.

## Introduction

 Ischemic heart disease is the leading cause of mortality worldwide. Despite advancements in medications and interventional cardiology, mortality has decreased in Western countries but not in developing countries.^1^ Early diagnosis of coronary artery disease (CAD) is crucial.

**Figure 1:**
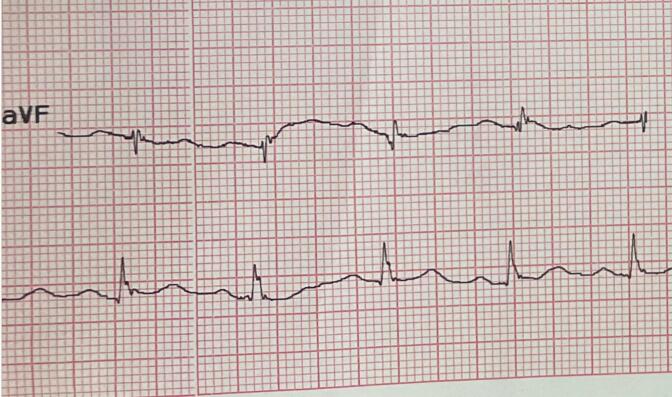
Fragmented QRS in lead II

 A fragmented QRS (fQRS) complex is defined as a notch in the R or S waves in leads with a QRS duration of less than 120 milliseconds and the absence of a bundle branch block. (Figure 1) The fQRS complex is caused by conduction abnormalities in the ventricle and disruption of ventricular depolarization secondary to myocardial ischemia or scar formation. It is an independent predictor of myocardial ischemia.^2^

 Traditionally, fQRS was considered a diagnostic tool for detecting myocardial infarction (MI)-related scar formation and predicting cardiac events and sudden cardiac deaths in patients with dilated cardiomyopathy.^3,4^ Nonetheless, recent studies have shown that ischemia itself, even without scar formation, can lead to fQRS.^2^

 The significance of fQRS among apparently healthy individuals was assessed in several studies. These investigations found that healthy subjects with fQRS had lower global longitudinal strain (GLS) values, even in the presence of normal ejection fraction. Consequently, fQRS was identified as a promising tool to identify individuals with regional left ventricular (LV) systolic dysfunction.^5,6^ GLS is a relatively novel method for detecting subtle LV dysfunction and serves as an earlier marker of LV dysfunction compared to ejection fraction.^7^

 Nevertheless, the association between reduced GLS and fQRS of myocardial segments and the localization of ischemic segments has not been evaluated thus far. In this study, we aimed to assess whether a combination of fQRS with strain echocardiography could identify coronary arteries with more than 70% stenosis.

## Materials and Methods

 This prospective case-control study was conducted at Dr Shariati Hospital between 2023 and 2024. The study population was composed of 110 patients referred to the cardiology department for ischemia evaluation, presenting with fQRS in their surface ECG. None of the patients had a history of MI or pathological Q waves in their ECGs, and all were in normal sinus rhythm. Patients with medical comorbidities, pericardial disease, and any type of cardiomyopathy, paced rhythm, or bundle branch block in surface ECG were excluded from the study. The Ethics Committee of Tehran University of Medical Sciences approved the study.

 Patients with typical chest pain and high probability for significant coronary artery stenosis underwent coronary angiography and for those with suspected coronary artery disease either single-photon emission computed tomography (SPECT) myocardial perfusion imaging or stress echocardiography were performed and in the case of inducible ischemia in theses noninvasive tests, coronary angiography was performed and otherwise medical management was planned and eventually based on the results of the tests, patients were divided into 2 subgroups: the ischemic group, consisting of patients with more than 70% stenosis in at least 1 major coronary artery, and the control group, comprising patients with normal coronary angiography, less than 30% stenosis of major coronary arteries, or those without conventional risk factors who had normal single-photon emission computed tomography (SPECT) myocardial perfusion imaging or stress echocardiography results.

 Demographic data, including age, sex, personal history of smoking or opium use, diabetes mellitus, dyslipidemia, and hypertension, were recorded.

 Patients were provided with sufficient information regarding the study objectives, and the following variables were collected through a questionnaire and echocardiography: LV end-systolic and diastolic sizes and volumes, E/E’ ratio, GLS and global circumferential strain values of 16 LV segments, and LV ejection fraction calculated via the Simpson method based on the American College of Cardiology/American Heart Association (ACC/AHA) guideline recommendations.^8^

 Following the collection of the aforementioned data, 2D speckle-tracking echocardiography was performed using automated cardiac motion quantification (aCMQ) with an EPIQ 7 (Philips Medical System, Andover, MA, USA) equipped with a 5-1 MHz sector transducer device in the ischemic and control groups.

 To quantify the 2D strain, 6 grayscale images were acquired and stored on digital media. Offline software was then used to generate the strain data. For the measurement of LV longitudinal strain, apical 4-, 2-, and 3-chamber views were imaged. Short-axis images at the basal, mid-ventricular, and apical views were obtained for the measurement of circumferential strain. An ECG was gated, and 3 cardiac cycles were recorded for each loop at between 30 and 70 frames per second. Automated border detection was used to trace the endocardium. Subsequently, the strain was automatically calculated by the software and expressed as a bull’s eye plot.

 Image acquisition and analysis of the samples were performed by a single echocardiographer blinded to the results of angiography. The same echocardiographer later reanalyzed the data to evaluate intra-observer variability.

###  Statistical analysis

 Data analysis was performed using SPSS version 22 software. Quantitative data were expressed as mean and standard deviation, while qualitative data were presented as numbers and percentages. Demographic and clinical variables of patients in the case and control groups were compared using t-tests and χ^2^ tests. Additionally, strain findings were compared between the 2 groups concerning fQRS using t-tests. A P value of less than 0.05 was considered statistically significant.

## Results

 In this study, we enrolled 55 patients with fQRS and more than 70% stenosis in at least 1 major coronary artery, as well as 55 patients with the same ECG pattern but less than 30% stenosis of coronary arteries or normal coronary arteries. As summarized in Table 1, there were no significant differences in baseline parameters, including mean age, body mass index, sex, history of diabetes mellitus, and cigarette, alcohol, and opium use, between the 2 groups. However, the incidence of hypertension was significantly higher in the group with significant coronary artery stenosis. The number of coronary vessels with and without significant stenosis is presented in Table 2.

**Table 1:** Basic Features of the Groups with and Without Significant Stenosis

**Variable**		**Case** **N=55**	**Control ** **N=55**	**P value**
Age, y		60/58 ± 10/44	61/45 ± 3/32	0/556
Body mass index, kg/m2		26/41 ± 6/46	25/64 ± 4/16	0/469
Sex	male	25(45/5)	26(47/3)	0/848
	female	30(54/5)	29(52/7)	
Hypertension		47(85/5)	36(65/5)	0/015
Diabetes mellitus		18(32/7)	12(24/8)	0/199
Cigarette smoking		13(23/6)	16/(29/1)	0/516
Opium use		1(1/8)	2(3/6)	0.558
Alcohol consumption		1(1/8)	5(9/1)	0.206

**Table 2:** Descriptions of Coronary Arteries with and without significant stenosis in the Case and Control Groups

**Involved Coronary Artery**		**Case**	**Control**	**All **
LAD	proximal	38(47/1)	31(50)	69(48/9)
	middle	34(43)	28(45/2)	62(44)
	distal	7(8/9)	3(4/8)	10(7/1)
	all	79(100)	62(100)	141(100)
LCX	proximal	29(82/9)	35(56/5)	64(66)
	middle	1(2/9)	2(3/2)	3(3/1)
	distal	5(14/3)	25(40/3)	30(30/9)
	all	35(100)	62(100)	97(100)
RCA	proximal	20(29)	26(39/4)	69(48/9)
	middle	27(39/1)	26(39/4)	62(44)
	distal	22(31/9)	14(21/2)	10(7/1)
	All	69(100)	64(100)	124(100)

LAD: left anterior descending artery, LCX: left circumflex artery, RCA: right coronary artery. The results are expressed as numbers (%).

Table 3 displays the ECG leads with fQRS. The leads were categorized based on the anatomic segments of the myocardium for subsequent comparison with the coronary artery that perfuses that segment and the strain of that segment.

**Table 3:** ECG Leads with fQRS in the Case and Control Groups

**ECG Lead**	**Case**	**Control**	**All**
V1, V2, and V3	9(16/4)	13(23/6)	22(20)
V4, V5, and V6	14(25/5)	12(21/8)	26(23/6)
II, III, and aVF/aVR	23(41/8)	24(43/6)	47(42/7)
I and aVL	8(14/5)	6(10/9)	14(12/7)
V5 and aVL	1(1/8)	0(0)	1(0/9)

The results are expressed as numbers (%).

Table 4 presents the strain values of different myocardial segments in patients with fQRS in ECG leads V1-V2-V3. The results indicated that the GLS in the basal anteroseptal wall was significantly lower in the case group (*P* = 0.028). Additionally, GLS in the apical inferior wall was significantly lower in the case group than in the control group (*P* < 0.0001).

**Table 4:** Strain Values of the Different Segments of the Myocardium in Patients With fQRS in Leads V1-V2-V3

**Strain Echocardiography**	**Segment**	**Case**	**Control**	**All**	**P value**
GLS					
Anteroseptal	basal	11/9 ± 5/67	17/8 ± 4/44	15/1 ± 5/67	0/028
	mid	10/5 ± 5/41	15/9 ± 6/7	13/6 ± 6/6	0/084
	apical	16/7 ± 6/06	18/2 ± 6/9	17/3 ± 6/4	0/494
Inferoseptal	basal	17/3 ± 6/4	19/3 ± 4/1	18/7 ± 4/7	0/488
	mid	18/2 ± 4/4	16/8 ± 3/5	16/8 ± 3/5	0/456
	apical	21/3 ± 5/2	16/2 ± 1/1	17/8 ± 3/7	0/093
Inferior	basal	17/1 ± 8/4	15/2 ± 5	15/9 ± 6/43	0/520
	mid	14/8 ± 5/5	15/58 ± 5/5	15/3 ± 5/4	0/757
GCS					
Anteroseptal	basal	16/7 ± 4/9	18/9 ± 4	18/1 ± 4/4	0.267
	mid	18/1 ± 8/9	18/8 ± 4	18/6 ± 6/2	0.811
	apical	16/4 ± 6/8	18/2 ± 4/8	17/5 ± 5/6	0.491
Inferior	base	16/7 ± 4/8	15/8 ± 11/5	16/2 ± 9/2	0/842
	mid	15/8 ± 10/7	19/4 ± 6/3	17/9 ± 8/3	0/362
	apical	15/2 ± 3/9	20/1 ± 8/5	18/2 ± 7/3	0/144

fQRS: fragmented QRS, GLS: global longitudinal strain, GCS: global circumferential strain. The results are expressed as %.

Table 5 demonstrates that GLS in patients with fQRS in leads V4-V5-V6 was significantly lower in the basal anteroseptal and basal anterior segments in the case group than in the control group (*P* = 0.009 and *P* = 0.042, respectively).

**Table 5:** Strain Values of the Different Segments of the Myocardium in Patients With fQRS in Leads V4-V5-V6

**Strain Echocardiography**	**Segment**	**Case**	**Control**	**All**	**P value**
GLS					
Anterior	basal	13/5 ± 5/4	18/5 ± 6/5	15/8 ± 6/4	0/042
	mid	13 ± 6	13/2 ± 4/9	13/1 ± 5/4	0/924
	apical	14/7 ± 3/6	14/9 ± 3/8	14/8 ± 3/6	0/928
Anteroseptal	basal	13/6 ± 6/1	19/5 ± 4/2	16/3 ± 5/8	0/009
	mid	16/6 ± 4/6	19/5 ± 2/2	17/9 ± 3/9	0/052
	apical	18/5 ± 3/5	19/5 ± 3/5	18/9 ± 3/4	0/524
GCS					
Anterior	basal	18/8 ± 8	18/8 ± 6	18/8 ± 7	0/892
	mid	19/4 ± 8/8	17/7 ± 6/7	18/6 ± 7/8	0/596
	apical	21/1 ± 10/4	16/7 ± 7/4	19/1 ± 9/2	0/264
Anteroseptal	basal	18/3 ± 5/8	19/2 ± 4/7	18/7 ± 5/3	0/687
	mid	19/4 ± 8/7	19/1 ± 7/9	19/3 ± 8/2	0/940
	apical	19/3 ± 10/7	19/1 ± 7/1	19/2 ± 9	0/961

fQRS: fragmented QRS, GLS: global longitudinal strain, GCS: global circumferential strain. The results are expressed as %.

Table 6 presents the strain values of different myocardial segments in patients with fQRS in ECG leads II, III, and aVF/aVR. The results demonstrated that GLS was significantly lower in the case group in the mid-inferoseptal and mid-inferior segments (*P* = 0.005 and *P* = 0.039, respectively).

**Table 6:** Strain Values of the Different Segments of the Myocardium in Patients With fQRS in Leads II-III-aVF/aVR

**Strain Echocardiography**	**Segment**	**Case**	**Control**	**All**	**P value**
GLS					
Inferoseptal	basal	15/1 ± 5/3	16/1 ± 4/8	15/6 ± 5	0/523
	mid	15/1 ± 3/2	19/5 ± 3/8	17 ± 3/9	0/005
	apical	19/4 ± 4/1	20/6 ± 3/4	20/3 ± 3/6	0/382
Inferior	basal	15/9 ± 4/8	14/6 ± 4/8	15/2 ± 4/8	0/353
	mid	4/3 ± 15/3	18/2 ± 4/5	16/7 ± 4/6	0/039
	apical	17/4 ± 5/3	19/6 ± 5/6	18/5 ± 5/5	0/223
Inferolateral	basal	16/2 ± 4/8	17/8 ± 9/1	17/2 ± 7/5	0/528
	mid	16/6 ± 4/5	18/6 ± 4/3	17/8 ± 4/4	0/183
	apical	19/3 ± 4/1	17/2 ± 4/7	18/1 ± 4/5	0/169
GCS					
Inferoseptal	basal	22 ± 6/2	19/4 ± 1/9	20/7 ± 4/7	0/069
	mid	20/8 ± 6/2	17/6 ± 9/1	19/2 ± 7/3	0/183
	apical	22/4 ± 7/7	18/1 ± 14/4	20/2 ± 11/7	0/230
Inferior	basal	20/5 ± 7/4	19/8 ± 2/7	20/1 ± 5/5	0/682
	mid	20/8 ± 6/7	17/1 ± 19/3	18/9 ± 8/3	0/143
	apical	23/2 ± 8/8	19/8 ± 4/8	21/5 ± 7/2	0/124
Inferolateral	basal	20/7 ± 7/1	19/4 ± 3/1	20/1 ± 5/5	0/421
	mid	20/8 ± 6/7	19/5 ± 2/9	20/1 ± 5/2	0/407
	apical	22/8 ± 9	21/2 ± 4/4	22 ± 7/1	0/467

fQRS: fragmented QRS, GLS: global longitudinal strain, GCS: global circumferential strain. The results are expressed as %.

Table 7 displays the strain values of different segments in patients with fQRS in ECG leads I and aVL. Based on data analysis, there were no significant differences in the strain values of all myocardial segments in this group.

**Table 7:** Strain Values of the Different Segments of the Myocardium in Patients With fQRS in Leads I and aVL

**Strain Echocardiography**	**Segment**	**Case**	**Control**	**All**	**P value**
GLS					
Antero lateral	basal	2.9 ± 13.4	14 ± 8.3	9.1 ± 11.6	0.357
	mid	3.9 ± 15.5	2.9 ± 14.2	3.4 ± 15	0.558
	apical	3.9 ± 16.4	4.9 ± 18	4.2 ± 17.1	0.558
Inferolateral	basal	3 ± 14.9	6.1 ± 12.9	4.4 ± 14	0.469
	mid	4.5 ± 15.6	4.8 ± 18.6	4.7 ± 16.8	0.288
	apical	2.7 ± 16.6	1.5 ± 17.6	2.2 ± 17	0.497
GCS					
Anterolateral	basal	5.1 ± 16.5	2.8 ± 19.1	4.6 ± 17.1	0.541
	mid	6.8 ± 17.8	1.8 ± 16.1	5.8 ± 17.4	0.738
	apical	7.3 ± 19.1	8.9 ± 15.7	7.2 ± 18.2	0.608
Inferolateral	basal	2.1 ± 17.9	4.7 ± 19.9	3.4 ± 18.7	0.335
	mid	6.4 ± 13.9	3.7 ± 19.7	6 ± 16.4	0.101
	apical	6 ± 18	4.9 ± 19.3	5.4 ± 18.5	0.704

fQRS: fragmented QRS, GLS: global longitudinal strain, GCS: global circumferential strain. The results are expressed as %.

## Discussion

 In the present study, we observed that in patients without a prior history of MI, the presence of fQRS on ECG in association with reduced segmental strain was predictive of significant coronary artery stenosis.

 While coronary angiography is the preferred method for evaluating both the scope and seriousness of CAD, its elevated cost, invasiveness, and radiation exposure hinder its regular application. As a result, other less-invasive techniques, such as stress echocardiography and thallium scans, have become standard semi-invasive tests for detecting coronary artery stenosis.

 Still, these diagnostic tests may cause significant complications, including sustained ventricular tachycardia, ventricular fibrillation, MI, any degree of atrioventricular block, and blood pressure fluctuations.^9,10^ Therefore, there is a need for alternative noninvasive tests that are safe, cost-effective, and free of complications. ECG is usually the first investigation performed for patients with suspected CAD, followed by echocardiography as the second most commonly requested test. When both tests yield normal results but clinical suspicion for CAD remains high, additional diagnostic modalities may be warranted. Echocardiographic strain imaging (deformation imaging) is a noninvasive and inexpensive method for assessing myocardial function. It does not involve radiation exposure and has gained popularity in clinical practice due to the significant limitation of ejection fraction, which only declines after substantial irreversible myocardial damage.^11^ Studies have shown that myocardial strain analysis using speckle-tracking echocardiography can accurately predict the severity of CAD.^12,13^ ECG is traditionally the primary modality used in the clinical evaluation of patients with suspected ischemia, but its sensitivity is limited when used alone. ST-segment changes, T-wave abnormalities, arrhythmias, and atrioventricular block are markers of ischemia.^14^ Numerous studies have demonstrated the association between fQRS in at least 2 contiguous leads on the 12-lead ECG with myocardial scarring from previous myocardial injury.^2,3-15^ Notably, fQRS reflects altered ventricular conduction and disrupted ventricular repolarization around the region of myocardial scarring.^16^

 Dosouty et al^17^ introduced the presence of fQRS as a useful predictor of significant CAD in patients presenting with acute coronary syndrome, even in those without enzyme elevation. The association between fQRS and ischemia in non-scarred myocardial segments has also been previously investigated.^2,18^ This abnormality in ischemic myocardium is thought to be related to inflammation at the cellular level.^19^ Additionally, Wijaya et al conducted a study evaluating the relationship between fQRS and the severity of coronary lesions, which demonstrated significant differences between mild-moderate and mild-severe Gensini scores.^20^

 Fragmentation of QRS has also been identified as a predictor of arrhythmic events in ischemic and dilated cardiomyopathy^21^ and serves as a marker of microvascular dysfunction.^22^ In patients with acute ST-elevation and non–ST-elevation MI, fQRS is associated with higher mortality, increased incidence of major adverse cardiovascular events, and reduced ejection fraction.^16,23^ Moreover, fQRS has been investigated in patients with various structural heart diseases, including ischemic and nonischemic cardiomyopathies, hypertrophic cardiomyopathy, valvular heart disease, sarcoidosis, Brugada syndrome, and arrhythmogenic right ventricular dysplasia, as well as in cases of inducible ventricular tachycardia.^24,25^ These findings suggest that fQRS reflects underlying myocardial disease with an arrhythmogenic substrate and serves as a predictive marker of higher cardiovascular disease mortality and future cardiac arrhythmias.

 While prior studies have supported the value of fQRS as a reliable predictor of myocardial scarring and mortality, some studies have reported contrasting findings. Wang et al^26^ retrospectively assessed 248 patients undergoing SPECT or coronary angiography and found that patients with significant left anterior descending artery disease were 3.680 times more likely to have fQRS; however, there was no association with major adverse cardiovascular events and all-cause mortality. In another study, MacAlpin et al^27^ investigated whether fQRS indicated a ventricular abnormality and discovered that, while this ECG abnormality was commonly associated with ventricular abnormalities, it could also occur in individuals without clinical heart disease. This finding may suggest that fQRS is an early sign of an arrhythmogenic right ventricular cardiomyopathy (ARVC) associated variant without an ARVC diagnosis. Nonetheless, its role in risk stratification within this subgroup remains limited.^28^

 Given that fQRS is not specific for ischemic myocardial damage and can be present in various cardiovascular diseases, additional imaging modalities may be required to increase its specificity. In our study, we combined echocardiographic strain imaging with fQRS to diagnose significant coronary artery stenosis, which was subsequently confirmed by coronary angiography. We found that patients with both fQRS in any ECG leads and reduced echocardiographic strain were more likely to have coronary arteries with over 70% stenosis. In contrast, Nikoo et al^5^ conducted a study in a healthy population with fQRS and reported reduced GLS values despite normal ejection fraction, suggesting regional LV systolic dysfunction. Nevertheless, coronary angiography or other imaging techniques were not employed in that study to rule out significant CAD.

## Conclusion

 In patients with CAD, the presence of fQRS in any ECG lead combined with reduced strain can predict the presence and location of a coronary artery with more than 70% stenosis.

## Competing Interests

 None.

## Ethical Approval

 The study was approved by the Ethics Committee of Tehran University of Medical Sciences (IR.Tums.THC.REC.1401.004).
